# Research advances in the application of vagus nerve electrical stimulation in ischemic stroke

**DOI:** 10.3389/fnins.2022.1043446

**Published:** 2022-10-28

**Authors:** Keling Cheng, Zhiyong Wang, Junhui Bai, Jie Xiong, Jianmin Chen, Jun Ni

**Affiliations:** Department of Rehabilitation, The First Affiliated Hospital of Fujian Medical University, Fuzhou, China

**Keywords:** vagus nerve stimulation, ischemic stroke, rehabilitation, upper limb dysfunction, dysphagia, cognitive dysfunction

## Abstract

Stroke seriously endangers human well-being and brings a severe burden to family and society. Different post-stroke dysfunctions result in an impaired ability to perform activities of daily living. Standard rehabilitative therapies may not meet the requirements for functional improvement after a stroke; thus, alternative approaches need to be proposed. Currently, vagus nerve stimulation (VNS) is clinically applied for the treatment of epilepsy, depression, cluster headache and migraine, while its treatment of various dysfunctions after an ischemic stroke is still in the clinical research stage. Recent studies have confirmed that VNS has neuroprotective effects in animal models of transient and permanent focal cerebral ischemia, and that its combination with rehabilitative training significantly improves upper limb motor dysfunction and dysphagia. In addition, vagus-related anatomical structures and neurotransmitters are closely implicated in memory–cognition enhancement processes, suggesting that VNS is promising as a potential treatment for cognitive dysfunction after an ischemic stroke. In this review, we outline the current status of the application of VNS (invasive and non-invasive) in diverse functional impairments after an ischemic stroke, followed by an in-depth discussion of the underlying mechanisms of its mediated neuroprotective effects. Finally, we summarize the current clinical implementation challenges and adverse events of VNS and put forward some suggestions for its future research direction. Research on VNS for ischemic stroke has reached a critical stage. Determining how to achieve the clinical transformation of this technology safely and effectively is important, and more animal and clinical studies are needed to clarify its therapeutic mechanism.

## Introduction

An ischemic stroke is an important social health problem worldwide and one of the leading causes of disability and death, severely impairing the quality of life of patients and imposing a serious economic burden on society. According to the 2020 World Stroke Organization (WSO) Declaration, the global stroke burden remains alarming, and if current trends continue, there will be nearly 200 million stroke survivors worldwide by 2050, with approximately 30 million new stroke patients each year thereafter (Brainin et al., 2020). When faced with an acute ischemic stroke, intravenous thrombolysis and mechanical thrombectomy are the main treatment options. Receiving the corresponding treatment within the appropriate time window can improve survival and reduce disability. However, only about 10% of patients with acute ischemic stroke are able to access rescue opportunity (Ma et al., 2019). Despite extensive advances in social medicine and scientific technology in recent years, nearly 60% of patients still have functional dysfunction within 6 months after a stroke (Lee et al., 2015). Therefore, it is paramount to propose a novel adjunctive therapy that can be clinically useful when acute stroke patients cannot be rescued in a timely manner and treatments to improve functional impairment in chronic stroke patients who do not meet their functional independence needs.

**FIGURE 1:**
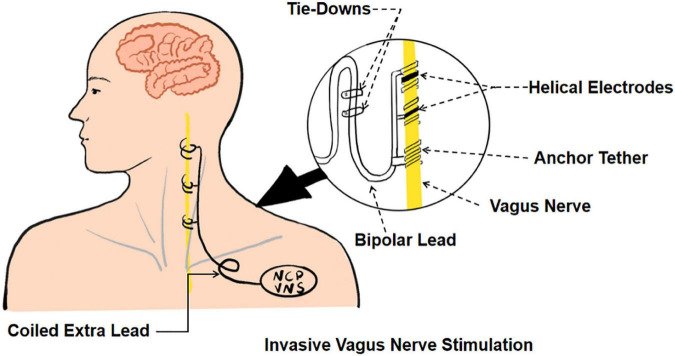
The schematic of invasive vagus nerve stimulation.

Invasive vagus nerve stimulation (iVNS) (see Figure 1) was approved by the United States Food and Drug Administration for the clinical treatment of refractory partial-onset epilepsy and patients suffering from severe recurrent unipolar and bipolar depression who failed to respond to at least four antidepressant interventions (Ben-Menachem, 2002; Carreno and Frazer, 2017). With the progress of research, the potential use of iVNS extends to a range of neurological disorders, such as upper limb, swallowing, and cognitive dysfunction after an ischemic stroke, traumatic brain injury, Parkinson’s disease, and Alzheimer’s disease (Khodaparast et al., 2013; Pruitt et al., 2016; Farrand et al., 2017; Vargas-Caballero et al., 2022).

**FIGURE 2:**
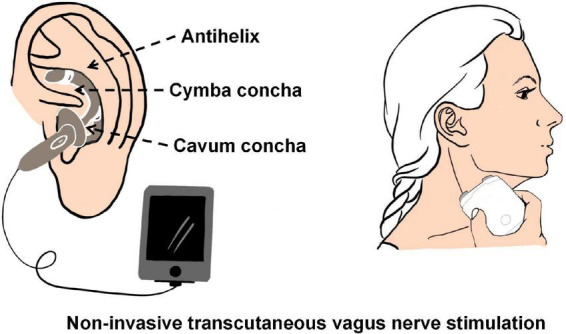
The schematic of transcutaneous cervical vagus nerve stimulation (tcVNS) and transcutaneous auricular vagus nerve stimulation (taVNS).

Although invasive vagus nerve electrical stimulation device implantation is a minimally invasive operation, it still carries potential surgical risks, such as respiratory abnormalities, vocal cord dysfunction, and peritracheal hematoma (Yap et al., 2020). Non-invasive transcutaneous vagus nerve stimulation techniques (tVNS) have been shown to activate vagal projections and vagal-mediated pathways similar to iVNS (Zhang et al., 2021b). tVNS is generally classified into two categories: transcutaneous cervical vagus nerve stimulation (tcVNS) and transcutaneous auricular vagus nerve stimulation (taVNS) (see Figure 2). Clear and repeatable vagal somatosensory-evoked potentials can be observed and recorded through electrical stimulation of the auricular branch of the vagus nerve and the cervical vagus nerve, which proves the feasibility of taVNS and tcVNS (Fallgatter et al., 2005; Nonis et al., 2017). In a rat model of acute cerebral ischemic injury, tcVNS resulted in a nearly 33% reduction in infarct volume and significantly improved neurological function scores and forelimb grip strength (Ay et al., 2016).

In this review, we first outline the current preclinical and clinical evidence for VNS (invasive and non-invasive) applied in upper limb motor, cognitive dysfunction, and dysphagia after an ischemic stroke and then delve into the potential mechanisms underlying the neuroprotective effects of VNS. We also discuss the side effects or adverse events of current VNS (invasive and non-invasive) technology in clinical practice. Finally, we summarize the remaining shortcomings of current VNS technology and suggest future improvements in clinical implementation and promising research directions.

## Application of vagus nerve stimulation in the rehabilitation of upper limb dysfunction after ischemic stroke

Upper limb dysfunction is one of the common sequelae of stroke, with approximately 75% of ischemic stroke patients still suffering from arm weakness after rehabilitation (Harvey and Nudo, 2007). Upper limb strength is also a predictor of arm function and a prognosis of chronic disability after a stroke (Harris and Eng, 2007). There is a large body of pre-clinical and clinical evidence showing that VNS combined with rehabilitation can promote the recovery of upper limb motor function after a stroke, but its safety and efficacy remain to be tested. In this section, we present some animal studies (Table 1) and clinical studies (Table 2) on invasive and non-invasive VNS techniques in the field of post-stroke upper limb dysfunction.

**TABLE 1:** Animal studies of VNS in post-stroke upper limb function.

References	Animal models	Device	Parameters	Stimulation site	Main findings
Porter et al., 2011	Adult female SD rats	Implantable VNS	30 Hz, 0.8 mA, 100 us, 500 ms, biphasic pulse	Left cervical Vagus nerve	Repeatedly VNS paired with a particular movement generate a specific increase in the motor cortex representation of that movement
Khodaparast et al., 2013	Adult female SD rats	Implantable VNS	30 Hz, 0.8 mA, 100 us, 500 ms, biphasic pulse	Left cervical Vagus nerve	VNS paired with rehabilitative training enhance recovery of forelimb force
Khodaparast et al., 2014	Adult female SD rats	Implantable VNS	30 Hz, 0.8 mA, 100 us, 500 ms, biphasic pulse	Left cervical Vagus nerve	Pairing VNS with rehabilitation can make the forelimb function recovered to pre-lesion level, while rehab training alone and delivering the same amount of stimulation after rehab fail to restore forelimb function to pre-lesion level
Hays et al., 2016	Female F344 rats, aged 18 months	Implantable VNS	30 Hz, 0.8 mA, 100 us, 500 ms, biphasic pulse	Left cervical Vagus nerve	VNS combined with rehabilitative training improves motor recovery after ischemic stroke in aged rats
Khodaparast et al., 2016	Female 4-month-old SD rats	Implantable VNS	30 Hz, 0.8 mA, 100 us, 500 ms, biphasic pulse	Left cervical Vagus nerve	VNS paired with rehabilitative training results in the significantly greater recovery of forelimb function in subjects with chronic ischemic stroke and may yield long-lasting benefits

**TABLE 2:** Clinical studies of VNS in post-stroke upper limb function.

References	Population	Stimulation site and device	VNS parameters	Paired	Main findings
Dawson et al., 2016	Chronic stroke, at least >6 months (*n* = 21)	Implantable VNS Left vagus nerve	Frequency 30 Hz Intensity 0.8 mA Pulse width 100 us Stimulation train 0.5 s Stimulation duration 3 times a week over 6 weeks	VNS paired with rehabilitation training	VNS paired with rehabilitation is feasible and without safety concerns
Kimberley et al., 2018	Chronic stroke, 4 months to 5 years (*n* = 17)	Implantable VNS Left vagus nerve	Frequency 30 Hz Intensity 0.8 mA Pulse width 100 us Stimulation train 0.5 s Stimulation duration 3 times a week over 6 weeks	VNS paired with upper limb rehabilitation, 6 weeks in-clinic therapy and 90 days at-home therapy	VNS combined with upper limb rehabilitation is associated with greater improvement in FMA-UE score at day 90 VNS paired with rehabilitation is acceptably safe and feasible in subject with chronic ischemic stroke
Dawson et al., 2020	Chronic stroke, 4 months to 5 years (*n* = 17)	Implantable VNS Left vagus nerve	Frequency 30 Hz Intensity 0.8 mA Pulse width 100 us Stimulation train 0.5 s	VNS paired with rehabilitation 6 weeks in-clinic therapy and 1-year follow up	Home-based VNS combined with rehabilitation is feasible and safe in subject with chronic stroke
Dawson et al., 2021	Chronic stroke, at least> 9 months (*n* = 108)	Implantable VNS Left vagus nerve	Frequency 30 Hz Intensity 0.8 mA Pulse width 100 us Stimulation train 0.5 s	VNS paired with rehabilitation 6 weeks in-clinic therapy and 90 days at-home therapy	VNS paired with rehabilitation is a novel potential strategy on people with long-term moderate-to-severe arm dysfunction after ischemic stroke
Capone et al., 2017	Ischemic or haemorrhagic chronic stroke (*n* = 14)	Left auricular vagus nerve Twister—EBM	Frequency 20 Hz Pulse width 0.3 ms Interval 30 s trains every 5 min Duration 60 min for 10 days Intensity above the detection threshold and below the pain threshold	taVNS delivered prior to robotic training Real or sham taVNS	taVNS combined with robotic rehabilitation is safe and tolerable and improve FMA scores significantly compared to the sham group after 10 days treatment
Redgrave et al., 2018b	Anterior circulation ischemic stroke >3 months (*n* = 13)	Left auricular vagus nerve Nemos (Cerbomed)	Frequency 25 Hz Pulse width 0.1 ms Duration 60 min 3 sessions per week for 6 weeks Median intensity 1.4 mA	taVNS paired with upper limb repetitive practice Single-group pre–post intervention study	taVNS combined with concurrent RTP is safe, feasible and associated with greater improvement in UFM score than RTP alone
Wu et al., 2020	Subacute ischemic stroke (*n* = 21)	Left auricular Vagus nerve BHD-1A transcutaneous electrical stimulation therapy instrument (Bohua, Weihai, China)	Frequency 20 Hz Pulse width 0.3 ms Mean stimulation intensity 1.66 mA Interval 30 s trains every 5 min Duration 60 min for 15 days	taVNS delivered prior to conventional rehabilitation training Real or sham taVNS	taVNS delivered prior to conventional rehabilitation is associated with greater improvement in UFM than sham group and its therapeutic effects are sustained at 12 weeks
Chang et al., 2021	Chronic stroke, at least >6 months (*n* = 36)	Left auricular Vagus nerve Self-designed	Frequency 30 Hz Pulse width 0.3 ms Intensity below pain threshold between 0.1 and 5.0 mA	taVNS delivered during robotic training Active or sham taVNS	taVNS combined with 3 weeks of upper limb robotic training showed significant reductions in spasticity at the wrist and hand and significant changes in sEMG peak amplitude

### Effects of vagus nerve stimulation on post-stroke upper limb dysfunction in rodent models

Porter et al. (2011) quantitatively assessed the effects of repeatedly pairing VNS with a specific movement on motor cortical plasticity through intracranial microstimulation and found that VNS paired with specific movements increased motor cortical representations, which was not a general effect of VNS but was specific to VNS paired with specific movements. Khodaparast et al. (2014) conducted a basic study separating 17 female stroke rats into three groups (VNS during rehabilitation, VNS after rehabilitation, and rehabilitation alone) and showed that VNS paired with rehabilitation significantly recovered forelimb function to prelesion level, while the other two groups failed to restore function to prelesion levels. This further validates the above findings that VNS combined with rehabilitation training may be a viable option for upper limb dysfunction after a stroke. The current clinical stroke population is predominantly middle-aged and elderly, with advanced age usually associated with a higher incidence of stroke and worse functional outcomes. A recent study reported that 6 weeks of paired VNS rehabilitation therapy in 18-month-old rats after ischemic lesions of the motor cortex, taking into account and integrating age factors, showed a significant increase in hit rate and peak pull forces within and between groups, suggesting the possibility of VNS paired with rehabilitative training in the treatment of elderly stroke patients (Hays et al., 2016). It is also noteworthy that VNS combined with rehabilitation therapy not only improves upper limb dysfunction in the acute phase of cerebral ischemia but also shows better therapeutic effects in the chronic phase of post-injury recovery compared to rehabilitation training alone. A study reported that VNS combined with rehabilitation training within 7–11 weeks after a cortical and subcortical ischemic lesion showed an 86% upper limb strength recovery compared to 47% for rehabilitation training alone and 42% for rehabilitative therapy with an equivalent VNS intervention delivered 2 h after daily rehabilitation training (Khodaparast et al., 2016).

### Effects of vagus nerve stimulation on post-stroke upper limb dysfunction in clinical trials

To better translate the results of animal studies and apply them to clinical practice, a large amount of clinical research evidence is still needed to support the feasibility and safety of VNS-paired rehabilitation training. In a randomized clinical controlled trial, 21 patients with chronic ischemic stroke (>6 months) were randomly assigned to VNS-paired rehabilitation training and rehabilitation alone groups, the findings of which showed some minor adverse effects (e.g., nausea and taste disturbance) and a significant difference in the Fugl–Meyer Upper Extremity Movement Score (FMA-UE) between the two groups after the intervention (Dawson et al., 2016). A blinded randomized multi-site clinical trial (iVNS/sham iVNS) investigating the potential effects of VNS combined with rehabilitation training to improve upper limb dysfunction in chronic stroke conducted 6 weeks of clinical treatment and 90 days of a home exercise program in patients with VNS implantation. The results showed no significant difference between the two groups after 6 weeks of clinical treatment, but there was a clinically significant difference in the FMA-UE and Wolf upper limb motor function ratings (WMFT) after 90 days of home training. Moreover, at the end of home training, the control group was crossed over to receive clinical rehabilitation paired with VNS and home training, and the results showed a significant increase in FMA-UE scores after 6 weeks of clinical intervention and 90 days of home training compared to the pre-crossover baseline level (Kimberley et al., 2018). Dawson et al. (2020) extended the follow-up period to 1 year to explore the long-term safety and feasibility of and adherence to home self-management (VNS combined with rehabilitation training) for patients with chronic stroke, showing that approximately 73% of the participants demonstrated a clinically meaningful improvement in FMA-UE at 1 year. Given the limitations of the above studies in terms of a small sample size and a non-blinded design, a subsequent study (pivotal, randomized, triple-blind, sham-controlled trial) was conducted by Dawson in 19 stroke rehabilitation services, fully supporting VNS-paired rehabilitation in the treatment of patients with upper limb motor dysfunction after chronic ischemic stroke (Dawson et al., 2021). Nevertheless, how this method can be applied in clinical rehabilitation to achieve the maximum effect and whether it can be used to improve more severe upper limb dysfunction are worthy of further study.

Concern about the potential risks of invasive procedures of iVNS techniques has prompted the rapid development and application of non-invasive VNS techniques in stroke. Capone et al. (2017) initially confirmed the feasibility and safety of taVNS combined with robotic rehabilitation in the treatment of patients with upper limb dysfunction after an ischemic stroke. Fourteen patients with ischemic or hemorrhagic chronic stroke were randomly assigned to two groups (robotic-assistive rehabilitation combined with true or sham taVNS training) for a 10-day intervention, showing no adverse events and a significant improvement in FMA scores in the real group compared to the sham group. Redgrave et al. (2018b) conducted a single-group, pre–post intervention study combining taVNS with repetitive task-specific practice (RTP) (1 h once, 3 times a week for 6 weeks). This pilot study found that patients’ UFM scores improved by an average of nearly 17 points compared to pre-intervention and that only three participants reported side effects (mild headache and fatigue), suggesting that taVNS combined with RTP is a feasible, safe, and tolerable treatment for upper limb weakness after a stroke (Redgrave et al., 2018b). To further investigate the effects and safety of taVNS-paired rehabilitation in upper limb dysfunction in patients with subacute ischemic stroke, Wu et al. (2020) selected 21 participants who were within 0.5–3 months of stroke onset and randomized them into conventional rehabilitation paired with real or sham taVNS stimulation. Significant improvements in the FMA-U, WMFT, and functional independence measurement (FIM) scores were observed in the real taVNS group compared to the sham-taVNS group, and taVNS appeared to be beneficial to the recovery of upper limb motor function in subacute ischemia stroke patients (Wu et al., 2020).

## Application of vagus nerve stimulation in the rehabilitation of dysphagia after ischemic stroke

Approximately 37%–78% of clinical acute stroke patients present with dysphagia, increasing the probability of complications, such as pneumonia, dehydration, and malnutrition, resulting in prolonged hospitalization and increased financial burden (Martino et al., 2005). VNS is a promising candidate for the treatment of stroke patients with dysphagia, partly because the innervation of the vagus nerve is closely related to swallowing and vocal cord movement, while the reduction of infarct volume and neuroprotective effects provides a relatively reliable guarantee of functional recovery after a stroke.

A case report on transcutaneous VNS for severe dysphagia after dorsal lateral medullary infarction showed that taVNS (current intensity: 2.5–3 mA, waveform: biphasic rectangular pulse, wave width: 500 ms, duration: 2 times a day, 5 days a week for 6 weeks) significantly reduced salivary residue and improved the ability of oral feeding, providing evidence for an in-depth investigation of vagus nerve electrical stimulation techniques for post-stroke dysphagia (Yuan et al., 2019). In addition, Long et al. (2022) conducted a preclinical study that provided stereological and immunohistochemical evidence of taVNS in an animal model of post-stroke dysphagia. taVNS was found to significantly increase the number of swallowing times within 20 s and the expression of vascular endothelial growth factor (VEGF) and basic fibroblast growth factor in white matter (Long et al., 2022). These findings raise the possibility of taVNS being an effective therapeutic strategy for the treatment of dysphagia after an ischemic stroke. It should be noted that vagus nerve magnetic stimulation has been initially applied in the treatment of post-stroke dysphagia in recent years. Lin et al. (2018) showed that repetitive transcranial magnetic stimulation could be an effective complementary treatment to traditional oropharyngeal rehabilitation. Nevertheless, there is still relatively scant clinical evidence of VNS for post-ischemic stroke dysphagia, and its effectiveness and safety remain to be proven.

## Application of vagus nerve stimulation in the rehabilitation of cognitive dysfunction after ischemic stroke

Improved cognitive function after a stroke is one of the basic requirements for the independent living ability of patients. One study found that, by using functional magnetic resonance imaging, invasive VNS caused changes in blood flow to brain regions, such as the thalamus, hippocampus, amygdala, brainstem, and hypothalamus, with the thalamus and amygdala being related to emotional memory and the hippocampus being associated with spatial and situational memory (King et al., 2002; Sucholeiki et al., 2002). Interestingly, the neuroprotective effects of VNS are partly exhibited by the activation of noradrenergic receptors mediated by the locus ceruleus, and the release of noradrenaline improves attention, responsiveness, and other cognitive functions. The underlying mechanism through which the stimulation of the vagus nerve enhances memory to improve cognitive processes is currently unknown, and it is speculated that the neurotransmitters associated with the vagus nerve could modulate this effect.

The VNS technology to improve cognitive function has mainly focused on healthy volunteers (Colzato et al., 2018) and epileptic patients (Martin et al., 2004). Colzato et al. (2018) administered a non-invasive tVNS to 80 healthy volunteers and showed a more pronounced creative performance in terms of divergent thinking, which could be associated with a transient increase in GABA concentration. McIntire et al. (2021) administered a non-invasive tcVNS to forty active-duty military participants under conditions of 34 h sleep deprivation to evaluate the efficacy of tcVNS as a fatigue countermeasure, and the results showed that participants receiving tcVNS performed significantly better on multi-tasking and arousal. In another study, epileptic patients underwent a left-sided vagus nerve electrical stimulation intervention (0.5 mA) using a gambling task to determine patient decision making, and the results showed that iVNS positively influenced patient decision making compared to a sham stimulation group (Martin et al., 2004). These studies provide evidence to explore the specific effects of VNS on cognitive dysfunction after an ischemic stroke.

Current studies on VNS to improve cognitive dysfunction after a stroke are mainly in the preclinical stage. Liu et al. (2016) administered iVNS (current intensity: 1 mA, frequency: 20 Hz, pulse width: 0.4 ms, stimulation duration and interval time: both 3 s) to an SD rat that had undergone middle cerebral artery occlusion (MACO) model and showed that VNS stimulation improved spatial and fear memory performance. In this particular study, the VNS-induced benefits in spatial and fear memory performance were reversed after the intracerebroventricular injection of DSP-4, which reduced norepinephrine (NE) levels in the cortical and hippocampal brain regions (Liu et al., 2016). The precise mechanism of VNS-induced cognitive improvement after a stroke is still not clearly elucidated, and the basic and clinical research evidence is still scanty.

## The underlying mechanism of vagus nerve stimulation in stroke

### Anti-inflammatory property

Researchers believe that VNS can exert a neuromodulatory effect to modulate systemic inflammatory responses through a broad vagal network (Yuan and Silberstein, 2016). This vagus nerve function is mainly mediated by three pathways, but it is still controversial. The first pathway is the splenic sympathetic anti-inflammatory pathway that modulates inflammation through the splenic sympathetic nerve, which releases norepinephrine linked to the β2 adrenergic receptor of splenic lymphocytes that release Ach. The second pathway is the anti-inflammatory hypothalamic–pituitary–adrenal axis, which is activated by vagal afferent fibers and prompts the adrenal glands to release cortisol. The third pathway is the vagal cholinergic anti-inflammatory pathway, a physiological mechanism through which vagal efferent fibers activate α7nAChR in peripheral macrophages via enteric neurons, modulating the systemic release of pro-inflammatory cytokines (Bonaz et al., 2016).

There is a growing body of evidence indicating that VNS exerts anti-inflammatory effects in ischemic stroke. Lu et al. (2017) reported that rats that had undergone PMACO received a7nAChR antagonist (A) and VNS treatment and found that the inhibition of a7nAchR attenuated the beneficial neuroprotective effects and the expression of a7nAchR, p-JAK2, and p-STAT3, indicating that VNS could suppress inflammation via the a7nAchR/JAK2 anti-inflammatory pathway. Li et al. (2020) found that the protein and mRNA expression levels of α7nAchR in the peri-infarct cortex examined by Western blotting, quantitative polymerase chain reaction (qPCR), and immunohistochemistry decreased 14 days after MCAO and that taVNS reversed the α7nAchR reduction. In addition, Liu et al. (2022) found that a significant decrease in the expression of cortical cyclooxygenase-2, calcitonin gene-related peptide in trigeminal ganglia and c-Fos in trigeminal nucleus caudalis after tcVNS administration, indicating that tcVNS also attenuated the cortical neuroinflammation.

There is a certain relationship between the activation of peroxisome proliferator-activated receptor gamma (PPAR-γ) and anti-inflammatory neuroprotective effects in various acute and chronic central nervous system (CNS) diseases (spinal cord injury, focal cerebral ischemia, etc.). One study found that VNS treatment enhanced PPAR-γ expression and inhibited pro-inflammatory cytokine expression and immune cell activation in the ischemic penumbra, suggesting that PPAR-γ could participate in the VNS-induced anti-inflammatory process (Jiang et al., 2015a).

### Inhabiting cell apoptosis and autophagy

The morphological and biochemical manifestations of apoptosis have been well investigated in experimental animal models of cerebral ischemia, especially in the ischemic penumbra and during reperfusion. Li et al. (1998) found that the ratio of apoptotic to necrotic cells was roughly 1:9, 1:6, and 1:13 in the ischemic core region and the inner and outer borders of the lesion, respectively. The cascade of apoptotic signaling is normally activated by both extrinsic and intrinsic apoptotic pathways after cerebral ischemia to regulate neuronal survival and death (Nakka et al., 2008). The caspases and Bcl-2 protein family, including pro-apoptotic proteins (Bax, Bad, Bak, etc.) and anti-apoptotic proteins (Bcl-2, Bcal-xL, etc.), are pivotal regulators of intracellular apoptotic signaling transduction. They not only play vital roles in regulating multiple apoptotic cell death pathways initiated during ischemia and reperfusion (I/R) but also suggest that targeted and potentially effective therapeutic strategies may be beneficial.

Jiang et al. (2014) determined the DNA fragmentation of apoptotic cells, cleaved caspase-3, and phosphorylated Akt (p-Akt) proteins using TUNEL staining and Western blot, respectively, in a rat model of focal cerebral I/R. This study found that VNS significantly decreased the level of TUNEL-positive cells and cleaved caspase-3 protein in ischemic penumbra, while p-Akt levels were significantly upregulated, suggesting that the neuroprotective effect of VNS is neuroprotective in acute cerebral I/R injury partly through the inhibition of apoptosis.

Following the study, the authors speculated that miR-210, an important microRNA regulated by hypoxia-inducible factor and Akt-dependent pathways, could be involved in the anti-apoptotic effects of VNS on I/R injury. In a rat model of MCAO, miR-210 expression was determined using real-time fluorescence qPCR, which revealed that VNS treatment enhanced miR-210 expression in ischemic stroke and that silencing miR-210 expression attenuated the VNS-induced improvement in infarct volume. To further investigate the specific role of miR-210 in the anti-apoptotic effect induced by VNS, the p-Akt protein and cleaved caspase-3 levels in rats preconditioned with miR-210 anticoagulant were determined using Western blot and immunofluorescence analysis, and the anti-apoptotic effect induced by VNS was found to be significantly attenuated following miR-210 knockdown, suggesting that miR-210 could be a protective factor that enhances the survival of brain tissue (Jiang et al., 2015b).

Autophagy, an alternative mode of cell death distinct from apoptosis, is closely associated with functional impairment in ischemic stroke. One study reported that VNS significantly downregulated the expression of Beclin-1 and decreased the LC3-II/LC3-I ratio compared to the I/R group, indicating that VNS exerts neuroprotective effects against I/R injury by inhibiting the autophagy pathway (Zhang et al., 2021a).

### Promoting angiogenesis and neuroprotection

Brain damage caused by decreased cerebral blood flow is the basic pathological process of ischemic stroke. Cerebral vascular remodeling plays a crucial role throughout the functional recovery phase of stroke, with improved collateral flow and a surge of angiogenesis being the main causes of increased cerebral blood volume (Liu et al., 2014). Given this, the hypothesis that VNS exerts a neuroprotective effect in ischemic stroke by increasing collateral blood flow and improving perfusion in the ischemic penumbra has been put forward. Nevertheless, evidence has suggested that the protective effect of VNS (iVNS and tVNS) in acute ischemic brain injury is not mediated by acute changes in focal cerebral blood flow and that an imbalance between maintaining tissue perfusion and autoregulatory vasodilation during ischemia could be responsible for the non-detection of subtle blood flow changes induced by VNS (Ay et al., 2011; Sun et al., 2012). In contrast, a study found that taVNS significantly increased microvessel density and endothelial cell proliferation surrounding the infarct area, indicating that taVNS enhanced the post-ischemic angiogenic response (Jiang et al., 2016). Recent studies have suggested that VNS-mediated angiogenesis after an ischemic stroke is associated with the expression of angiogenic factors, such as brain-derived neurotrophic factor (BDNF), VEGF, and growth differentiation factor-11 (GDF11) (Jiang et al., 2016; Ma et al., 2016, 2018).

Brain-derived neurotrophic factor and VEGF are involved in and contribute to the development of angiogenesis. Silencing VEGF expression during the developmental stage of the brain can lead to impaired blood vessel formation. Jiang et al. (2016) used double immunofluorescence staining and Western blot to analyze BDNF and VEGF proteins and mRNA expressions and found that taVNS significantly upregulated BDNF and VEGF proteins and mRNA levels at the border of the ischemic zone after 21 days of reperfusion.

In addition, as GDF11 possesses the ability to participate in vascular remodeling, improve cerebral vascular function, increase neurogenesis, and enhance the proliferation of primary brain capillary endothelial cells (ECs) (Katsimpardi et al., 2014), speculation has been raised as to whether GDF11 mediates the potential mechanism of VNS angiogenesis. Ma et al. (2016) performed taVNS intervention in a rat model of MCAO and examined GDF11 protein and mRNA expression in the brain and spleen. This study found that taVNS promoted EC proliferation and activin-like kinase 5 (ALK5) expression, upregulated brain GDF11, and downregulated spleen GDF11, suggesting that GDF11 could be involved in taVNS-mediated angiogenic mechanisms through the ALK5 pathway. Following the above study, the authors again elaborated in 2018 that GDF11/ALK5 is involved in and promotes the mechanism of taVNS angiogenesis and is expected to be a new therapeutic target for stroke rehabilitation (Ma et al., 2018).

### Protecting the integrity of the blood–brain barrier

The blood–brain barrier (BBB) plays an important role in regulating blood, solute, and cell transport at the blood–brain interface and maintaining the homeostatic microenvironment of the CNS. Ischemic stroke leads to inflammatory damage of endothelial cells and increased permeability of paracellular and transcellular pathways, resulting in damage to the BBB, which further promotes the entry of liquids, chemicals, and blood-derived cells into the brain parenchyma, causes brain edema, and aggravates inflammatory response and brain injury (Jiang et al., 2018). A study found that IL-17 could disrupt the integrity of the BBB and increase the production of reactive oxygen species (Huppert et al., 2010). However, non-invasive cervical vagus nerve electrical stimulation can promote microglia M2 polarization, mediating anti-I/R brain injury by inhibiting IL-17 levels. This suggests that the complex relationship between the VNS inhibition of IL-17 and the BBB in a model of cerebral ischemia needs further elucidation (Zhao et al., 2019).

Yang et al. (2018) found that tcVNS significantly decreased the BBB transfer rate in the lesion area and reduced the expression of matrix metalloproteinases-2/9 in reactive astrocytes in the ischemic hemisphere. In addition, a study investigating the effect of VNS on cortical microinfarction in mice with or without colitis showed that VNS decreased BBB permeability and protected BBB integrity using two-photon imaging (Chen et al., 2018).

### Reducing spreading depolarization

Cortical spreading depression (CSD) is a slowly intensive depolarization wave of neurons and glial cells propagating across the cerebral gray matter. Spreading depolarizations (SDs) are one of the key mechanisms of cerebral ischemic injury, and they usually refer to waves of abrupt, sustained mass depolarization in the gray matter of the CNS (Dreier and Reiffurth, 2015). The depolarization of ischemic neurons and glial cells is due to an insufficient energy supply and the release of glutamate and potassium ions (Gavaret et al., 2019). SDs can induce cytotoxic edema, and although this toxic state is reversible in the early stage of injury, SD-related low perfusion can cause spreading ischemia, which further aggravates brain injury (Dreier et al., 2018). Lindemann et al. (2020) used a rat model of PMACO to explore whether VNS could be used as a new non-pharmacological means to inhibit local SDs and reduce the risk of stroke. Compared to sham VNS stimulation, invasive VNS or non-invasive VNS stimulation was found to significantly reduce the frequency of SDs in the peri-infarcted area, but the amplitude and velocity remained unchanged, and VNS had no significant effect on pulse, respiratory frequency, or blood oxygen saturation during stimulation (Lindemann et al., 2020). They postulated that VNS could be a safe and effective intervention to reduce the clinical burden of SD waves in stroke patients. In another study, Chen et al. (2016) found that both iVNS and tcVNS significantly inhabits CSD susceptibility in the occipital cortex in rats, and this effect occurred within 30 min after vagus nerve stimulation and lasts for more than 3 h. Morais et al. (2020) investigated the central and peripheral mechanisms underlying iVNS and tcVNS efficacy on CSD and found that CSD suppression by VNS is mediated by activation of the vagal visceral sensory afferents relaying in nucleus tractus solitarius and in turn projecting to subcortical neuromodulatory centers. To further determine the optimal tcVNS paradigm of CSD suppression, Liu et al. (2022) tested the efficacy of various intensities and doses of tcVNS on CSD frequency and electrical threshold of CSD and found that two 2-min nVNS 5 min apart afforded the highest efficacy on electrical CSD threshold and frequency of CSD, suggesting that tcVNS inhabits CSD susceptibility in an intensity-dependent manner. Given the importance of the spreading depolarization mechanism in the physiological and pathological processes of ischemic stroke and the lack of sufficient research evidence to elucidate the specific effects of VNS on SDs in rodent models of cerebral ischemia, this field remains to be further explored.

## Side effects of vagus nerve stimulation

The safety of invasive VNS has always been a key indicator of whether this technique can be successfully applied in clinical practice. The complications of iVNS treatment are mainly related to surgical procedures and nerve stimulation. A retrospective, single-center, longitudinal study found that 16.8% of 143 patients who underwent 251 iVNS procedures had surgery-related complications, and 16.8% of the complications were related to hardware malfunctions (lead fracture, disconnection, spontaneous turn-off, and stimulator malfunction) (Kahlow and Olivecrona, 2013). It should be noted that the replacement of stimulators can also increase the risk of infection, and the service life of the equipment should be considered as one of the factors for patients undergoing long-term iVNS treatment.

Bradycardia is potentially one of the major complications during the initial iVNS procedure, requiring cardiac resuscitation, even in the absence of pre-existing cardiovascular disease (Fahy, 2010). One study recorded three patients who showed bradycardia during the intraoperative VNS Lead Test in 111 subjects, but the symptoms disappeared spontaneously after the operation (Ardesch et al., 2007). To better locate the vagus nerve during the operation, neck dissection may cause tracheal injury or pharyngeal dysfunction. Surgery-related complications also include neck pain, wound infection, peritracheal hematoma, and temporary vocal cord paralysis, but the average incidence of surgical complications in iVNS is less than 5% (Toffa et al., 2020).

After iVNS, the adverse reactions of patients were mainly related to nerve stimulation, including temporary vocal cord paralysis, mild hoarseness, dyspnea, and cough (Rychlicki et al., 2006). VNS stimulation can also induce sleep apnea syndrome, which is a reasonable routine screening before and after VNS implantation (Salvade et al., 2018). The above summarizes the common side effects of the iVNS intervention. Some less common side effects have also been documented, including delayed hoarseness, permanent left vocal cord paralysis, sternocleidomastoid spasm, and shortness of breath (Rychlicki et al., 2006; Tran et al., 2011).

As a potential alternative therapy for iVNS, it is critical to record and summarize the type, severity, and incidence of related adverse events or side effects of tVNS. A review by Silberstein et al. (2020) reported that the most common adverse device events (ADEs) of tVNS are mainly related to the application site discomfort (4.6%), application site irritation/redness (3.4%), local pain in face/head/neck area (2.8%), muscle twitching and/or contractions in face/head/neck area (2.7%), headache/migraine (2.6%), dizziness (2.0%) and tingling/prickling on the skin (1.7%).

Redgrave et al. (2018a) showed a significant relationship between tVNS side effect rate and stimulus frequency (the frequency of one study is four times that of other studies) and no significant relationship between pulse width and rate of side effects. However, due to the lack of detailed parameter information in each study, it is impossible to analyze the independent influence of the side effect rate using multivariate linear regression. The relationship between the VNS side effect rate and treatment dose is still unclear, which should be a priority of future research, and detailed documentation of the incidence of each adverse event can contribute to the further elucidation of the potential association between the two. Morrison et al. (2019) examined the effects of different intensities of VNS (0.4, 0.8, and 1.6 mA) combined with upper limb rehabilitation on motor cortical plasticity and found that moderate-intensity VNS combined with rehabilitation VNS produced more significant improvements than the other two using intracortical microstimulation. The following two understandings exist regarding the diminished therapeutic effect associated with increased VNS stimulation. First, there is an inverted U-shaped relationship between the intensity of VNS stimulation and the therapeutic effect, which may be related to the desensitization of increased VNS stimulation. Although the specific mechanism is not clear, a variety of cellular mechanisms are possible. Second, additional VNS stimulation may enhance non-specific movements during non-specific motor tasks, resulting in competitive interference.

## Current barriers and future prospects

### Adjusting stimulation parameters

Setting and adjusting the optimal electrical stimulation parameters have always been the most critical challenges related to the clinical application and efficacy of VNS. To determine the stimulation paradigm that produces the greatest effect of VNS therapy, Hays et al. (2014) investigated the effect of VNS stimulation timing and amount in terms of treatment efficacy, and their findings showed that similar amounts of delayed VNS delivered 2 h after daily rehabilitative training and several-fold more VNS failed to improve recovery to the same degree as VNS, which is timed to occur with successful movements.

The setting of the current intensity and pulse width is crucial when it comes to VNS stimulation parameters. When the current intensity is adjusted and moderately increased, the release of neurotransmitters and the discharge rate of locus coeruleus cells gradually increase (Roosevelt et al., 2006; Hulsey et al., 2017). At the same time, under the same current intensity, appropriately increasing the pulse width can enhance the therapeutic effect of VNS (Loerwald et al., 2018). Most studies have set the VNS frequency to 20–30 Hz, not only in iVNS but also in tVNS.

Finally, it is not clear whether there is a direct correlation between the parameter setting of VNS stimulation and VNS adverse reactions or clinical side effects, and more clinical evidence is needed to clarify the relationship between them. Taking into account the individual differences of the subjects, some subjects may have an excessive or no stimulus response to the inherent parameters of the current VNS stimulation device. It also has a reference value to formulate personalized parameters combined with patients’ pain threshold or sensory threshold. To date, the safest and most effective combination of VNS parameter settings has not been determined; therefore, it is vital to continuously optimize and adjust VNS stimulation parameters.

### Optimizing animal models

In preclinical animal models of cerebral ischemic stroke, the neuroprotective effects of iVNS and tVNS technologies have been proven. Considering that the current animal models of VNS ischemic stroke still have limitations with regard to clinical practice, it is important to meet the criteria for preclinical recommendations of the Stroke Treatment Academic Roundtable for the treatment of acute stroke (Fisher et al., 2009).

First, the majority of studies used partial or total middle cerebral artery occlusion animal models, and even though a small number of studies used posterior circulation injury models, they had low reproducibility. Therefore, the embolization site, especially small vessel occlusion and posterior circulation infarction, should be fully taken into account when constructing animal models of VNS cerebral ischemia.

Second, studies have mostly used transient MACO and reperfusion models, while patients are not able to access timely medical help for urgent revascularization (mechanical retrieval and intravenous thrombolysis). Therefore, further studies on permanent vascular occlusion models are important to recapitulate a common stroke phenotype in clinical practice.

Third, the study reported that the molecular mechanisms leading to ischemic cell death differ in the two sexes (Manwani and McCullough, 2011). Given sexual dimorphism in ischemic stroke, there is a need to administer VNS in ischemic stroke studies in male and female animal models, providing strong and reliable evidence for clinical transformation.

Fourth, the occurrence of human stroke may be the result of multiple factors, such as hypertension, aging, diabetes, heart disease, and medications. It has been proven that preclinical studies of acute stroke treatment, predominantly in young and healthy animals, have low external validity, which indicates an overestimation of stroke treatment efficacy in experimental studies (Schmidt-Pogoda et al., 2020). There is a need to conduct studies in animals with comorbidities that are a common phenotype of stroke patients, given that the clinical stroke population is predominantly older adults with comorbidities.

Fifth, most current preclinical studies mainly involve rodents, but in the future, advanced experimental animals, such as cats or primates, can be used for research. Although no studies have directly demonstrated their experimental advantages, the anatomical structure of the brain, which is more similar to that of humans, may be more convincing.

### Auricular vs. cervical stimulation

There is still no direct comparison between taVNS and tcVNS in either animal models or clinical studies of cerebral ischemic stroke. It should be noted that the optimal stimulation parameters for taVNS and tcVNS are not yet uniformly defined before a direct comparison between the two, and it is crucial to optimize the stimulation parameters and perform a comparison of efficacy in the context of a range of different parameters. Ay et al. (2016) studied the protective effect of tVNS on ischemic stroke using taVNS and tcVNS techniques in 2015 and 2016, respectively, and found that tcVNS (28.75 ± 4.22%) showed a higher reduction in infarct size than taVNS (31.65 ± 9.67%). It is important to mention that the animal models used in these two studies were different (Wistar rats and spontaneously hypertensive rats, respectively) (Ay et al., 2015, 2016). Aside from the differences in efficacy between taVNS and tcVNS, the concern of safety is also worthy of attention. The preference of the two treatment modalities for different degrees of brain injury or for different age groups of patients deserves further discussion.

## Conclusion

To date, there is a large body of evidence supporting the clinical application value of vagus nerve electrical stimulation in improving post-stroke dysfunctions (upper limb movement, swallowing, and cognition). Although the exact mechanism of the neuroprotective effect of VNS is not yet conclusive, we are currently in a critical period of clinical VNS application of value transformation. During this phase, it is necessary to document in detail and correct the efficacy and adverse events of VNS clinical applications, optimize and adjust animal research models and stimulation parameters, develop innovative stimulation devices, and expand the indications of VNS clinical practice.

## Author contributions

JN and KC conceived and organized the writing of the manuscript. KC, ZW, and JB researched literature and wrote the manuscript. KC, ZW, and JX proofread the writing of the manuscript. All authors contributed to the article and approved the submitted version.

## References

[B1] Ardesch J. J. Buschman H. P. van der Burgh P. H. Wagener-Schimmel L. J. van der Aa H. E. Hageman G. (2007). Cardiac responses of vagus nerve stimulation: Intraoperative bradycardia and subsequent chronic stimulation. *Clin. Neurol. Neurosurg.* 109 849–852. 10.1016/j.clineuro.2007.07.024 17825483

[B2] Ay I. Napadow V. Ay H. (2015). Electrical stimulation of the vagus nerve dermatome in the external ear is protective in rat cerebral ischemia. *Brain Stimul.* 8 7–12. 10.1016/j.brs.2014.09.009 25312600 PMC4277719

[B3] Ay I. Nasser R. Simon B. Ay H. (2016). Transcutaneous Cervical Vagus Nerve Stimulation Ameliorates Acute Ischemic Injury in Rats. *Brain Stimul.* 9 166–173. 10.1016/j.brs.2015.11.008 26723020 PMC4789082

[B4] Ay I. Sorensen A. G. Ay H. (2011). Vagus nerve stimulation reduces infarct size in rat focal cerebral ischemia: An unlikely role for cerebral blood flow. *Brain Res.* 1392 110–115. 10.1016/j.brainres.2011.03.060 21458427 PMC3293221

[B5] Ben-Menachem E. (2002). Vagus-nerve stimulation for the treatment of epilepsy. *Lancet Neurol.* 1 477–482. 10.1016/s1474-4422(02)00220-x 12849332

[B6] Bonaz B. Sinniger V. Pellissier S. (2016). Anti-inflammatory properties of the vagus nerve: Potential therapeutic implications of vagus nerve stimulation. *J. Physiol.* 594 5781–5790. 10.1113/JP271539 27059884 PMC5063949

[B7] Brainin M. Feigin V. L. Norrving B. Martins S. C. O. Hankey G. J. Hachinski V. (2020). Global prevention of stroke and dementia: The WSO Declaration. *Lancet Neurol.* 19 487–488. 10.1016/s1474-4422(20)30141-1 32470419

[B8] Capone F. Miccinilli S. Pellegrino G. Zollo L. Simonetti D. Bressi F. (2017). Transcutaneous Vagus Nerve Stimulation Combined with Robotic Rehabilitation Improves Upper Limb Function after Stroke. *Neural Plast.* 2017:7876507. 10.1155/2017/7876507 29375915 PMC5742496

[B9] Carreno F. R. Frazer A. (2017). Vagal Nerve Stimulation for Treatment-Resistant Depression. *Neurotherapeutics* 14 716–727. 10.1007/s13311-017-0537-8 28585221 PMC5509631

[B10] Chang J. L. Coggins A. N. Saul M. Paget-Blanc A. Straka M. Wright J. (2021). Transcutaneous auricular vagus nerve stimulation (tAVNS) delivered during upper limb interactive robotic training demonstrates novel antagonist control for reaching movements following stroke. *Front. Neurosci*. 15:767302. 10.3389/fnins.2021.767302 34899170 PMC8655845

[B11] Chen S. P. Ay I. Lopes de Morais A. Qin T. Zheng Y. Sadeghian H. (2016). Vagus nerve stimulation inhibits cortical spreading depression. *Pain* 157 797–805. 10.1097/j.pain.0000000000000437 26645547 PMC4943574

[B12] Chen X. He X. Luo S. Feng Y. Liang F. Shi T. (2018). Vagus Nerve Stimulation Attenuates Cerebral Microinfarct and Colitis-induced Cerebral Microinfarct Aggravation in Mice. *Front. Neurol.* 9:798. 10.3389/fneur.2018.00798 30319530 PMC6168656

[B13] Colzato L. S. Ritter S. M. Steenbergen L. (2018). Transcutaneous vagus nerve stimulation (tVNS) enhances divergent thinking. *Neuropsychologia* 111 72–76. 10.1016/j.neuropsychologia.2018.01.003 29326067

[B14] Dawson J. Engineer N. D. Prudente C. N. Pierce D. Francisco G. Yozbatiran N. (2020). Vagus Nerve Stimulation Paired With Upper-Limb Rehabilitation After Stroke: One-Year Follow-up. *Neurorehabil. Neural Repair* 34 609–615. 10.1177/1545968320924361 32476617

[B15] Dawson J. Liu C. Y. Francisco G. E. Cramer S. C. Wolf S. L. Dixit A. (2021). Vagus nerve stimulation paired with rehabilitation for upper limb motor function after ischaemic stroke (VNS-REHAB): A randomised, blinded, pivotal, device trial. *Lancet* 397 1545–1553. 10.1016/s0140-6736(21)00475-x 33894832 PMC8862193

[B16] Dawson J. Pierce D. Dixit A. Kimberley T. J. Robertson M. Tarver B. (2016). Safety, Feasibility, and Efficacy of Vagus Nerve Stimulation Paired With Upper-Limb Rehabilitation After Ischemic Stroke. *Stroke* 47 143–150. 10.1161/STROKEAHA.115.010477 26645257 PMC4689175

[B17] Dreier J. P. Reiffurth C. (2015). The stroke-migraine depolarization continuum. *Neuron* 86 902–922. 10.1016/j.neuron.2015.04.004 25996134

[B18] Dreier J. P. Lemale C. L. Kola V. Friedman A. Schoknecht K. (2018). Spreading depolarization is not an epiphenomenon but the principal mechanism of the cytotoxic edema in various gray matter structures of the brain during stroke. *Neuropharmacology* 134 189–207. 10.1016/j.neuropharm.2017.09.027 28941738

[B19] Fahy B. G. (2010). Intraoperative and perioperative complications with a vagus nerve stimulation device. *J. Clin. Anesth.* 22 213–222. 10.1016/j.jclinane.2009.10.002 20400010

[B20] Fallgatter A. J. Ehlis A. C. Ringel T. M. Herrmann M. J. (2005). Age effect on far field potentials from the brain stem after transcutaneous vagus nerve stimulation. *Int. J. Psychophysiol.* 56 37–43. 10.1016/j.ijpsycho.2004.09.007 15725488

[B21] Farrand A. Q. Helke K. L. Gregory R. A. Gooz M. Hinson V. K. Boger H. A. (2017). Vagus nerve stimulation improves locomotion and neuronal populations in a model of Parkinson’s disease. *Brain Stimul.* 10 1045–1054. 10.1016/j.brs.2017.08.008 28918943 PMC5675746

[B22] Fisher M. Feuerstein G. Howells D. W. Hurn P. D. Kent T. A. Savitz S. I. (2009). Update of the stroke therapy academic industry roundtable preclinical recommendations. *Stroke* 40 2244–2250. 10.1161/STROKEAHA.108.541128 19246690 PMC2888275

[B24] Gavaret M. Marchi A. Lefaucheur J. P. (2019). Clinical neurophysiology of stroke. *Handb. Clin. Neurol.* 161 109–119. 10.1016/B978-0-444-64142-7.00044-8 31307595

[B25] Harris J. E. Eng J. J. (2007). Paretic Upper-Limb Strength Best Explains Arm Activity in People With Stroke. *Phys. Ther.* 87 88–97. 10.2522/ptj.20060065 17179441

[B26] Harvey R. L. Nudo R. J. (2007). Cortical brain stimulation: A potential therapeutic agent for upper limb motor recovery following stroke. *Top. Stroke Rehabil.* 14 54–67. 10.1310/tsr1406-54 18174116

[B27] Hays S. A. Khodaparast N. Ruiz A. Sloan A. M. Hulsey D. R. Rennaker R. L. II (2014). The timing and amount of vagus nerve stimulation during rehabilitative training affect poststroke recovery of forelimb strength. *Neuroreport* 25 676–682. 10.1097/WNR.0000000000000154 24818637 PMC4039714

[B28] Hays S. A. Ruiz A. Bethea T. Khodaparast N. Carmel J. B. Rennaker R. L. II (2016). Vagus nerve stimulation during rehabilitative training enhances recovery of forelimb function after ischemic stroke in aged rats. *Neurobiol. Aging* 43 111–118. 10.1016/j.neurobiolaging.2016.03.030 27255820 PMC5206764

[B29] Hulsey D. R. Riley J. R. Loerwald K. W. Rennaker R. L. II Kilgard M. P. Hays S. A. (2017). Parametric characterization of neural activity in the locus coeruleus in response to vagus nerve stimulation. *Exp. Neurol.* 289 21–30. 10.1016/j.expneurol.2016.12.005 27988257 PMC5297969

[B30] Huppert J. Closhen D. Croxford A. White R. Kulig P. Pietrowski E. (2010). Cellular mechanisms of IL-17-induced blood-brain barrier disruption. *FASEB J.* 24 1023–1034. 10.1096/fj.09-141978 19940258

[B31] Jiang X. Andjelkovic A. V. Zhu L. Yang T. Bennett M. V. L. Chen J. (2018). Blood-brain barrier dysfunction and recovery after ischemic stroke. *Prog. Neurobiol.* 16 144–171. 10.1016/j.pneurobio.2017.10.001 28987927 PMC5886838

[B32] Jiang Y. Li L. Liu B. Zhang Y. Chen Q. Li C. (2014). Vagus nerve stimulation attenuates cerebral ischemia and reperfusion injury via endogenous cholinergic pathway in rat. *PLoS One* 9:e102342. 10.1371/journal.pone.0102342 25036185 PMC4103831

[B33] Jiang Y. Li L. Liu B. Zhang Y. Chen Q. Li C. (2015a). PPARgamma upregulation induced by vagus nerve stimulation exerts anti-inflammatory effect in cerebral ischemia/reperfusion rats. *Med. Sci. Monit.* 21 268–275. 10.12659/MSM.891407 25619160 PMC4310716

[B34] Jiang Y. Li L. Ma J. Zhang L. Niu F. Feng T. (2016). Auricular vagus nerve stimulation promotes functional recovery and enhances the post-ischemic angiogenic response in an ischemia/reperfusion rat model. *Neurochem. Int.* 97 73–82. 10.1016/j.neuint.2016.02.009 26964767

[B35] Jiang Y. Li L. Tan X. Liu B. Zhang Y. Li C. (2015b). miR-210 mediates vagus nerve stimulation-induced antioxidant stress and anti-apoptosis reactions following cerebral ischemia/reperfusion injury in rats. *J. Neurochem.* 134 173–181. 10.1111/jnc.13097 25783636

[B36] Kahlow H. Olivecrona M. (2013). Complications of vagal nerve stimulation for drug-resistant epilepsy: A single center longitudinal study of 143 patients. *Seizure* 22 827–833. 10.1016/j.seizure.2013.06.011 23867218

[B37] Katsimpardi L. Litterman N. K. Schein P. A. Miller C. M. Loffredo F. S. Wojtkiewicz G. R. (2014). Vascular and neurogenic rejuvenation of the aging mouse brain by young systemic factors. *Science* 344 630–634. 10.1126/science.1251141 24797482 PMC4123747

[B38] Khodaparast N. Hays S. A. Sloan A. M. Fayyaz T. Hulsey D. R. Rennaker R. L. II (2014). Vagus nerve stimulation delivered during motor rehabilitation improves recovery in a rat model of stroke. *Neurorehabil. Neural Repair* 28 698–706. 10.1177/1545968314521006 24553102 PMC4134702

[B39] Khodaparast N. Hays S. A. Sloan A. M. Hulsey D. R. Ruiz A. Pantoja M. (2013). Vagus nerve stimulation during rehabilitative training improves forelimb strength following ischemic stroke. *Neurobiol. Dis.* 60 80–88. 10.1016/j.nbd.2013.08.002 23954448

[B40] Khodaparast N. Kilgard M. P. Casavant R. Ruiz A. Qureshi I. Ganzer P. D. (2016). Vagus Nerve Stimulation During Rehabilitative Training Improves Forelimb Recovery After Chronic Ischemic Stroke in Rats. *Neurorehabil. Neural Repair* 30 676–684. 10.1177/1545968315616494 26542082 PMC4854844

[B41] Kimberley T. J. Pierce D. Prudente C. N. Francisco G. E. Yozbatiran N. Smith P. (2018). Vagus Nerve Stimulation Paired With Upper Limb Rehabilitation After Chronic Stroke. *Stroke* 49 2789–2792. 10.1161/STROKEAHA.118.022279 30355189

[B42] King J. A. Burgess N. Hartley T. Vargha-Khadem F. O’Keefe J. (2002). Human hippocampus and viewpoint dependence in spatial memory. *Hippocampus* 12 811–820. 10.1002/hipo.10070 12542232

[B44] Lee K. B. Lim S. H. Kim K. H. Kim K. J. Kim Y. R. Chang W. N. (2015). Six-month functional recovery of stroke patients: A multi-time-point study. *Int. J. Rehabil. Res.* 38 173–180. 10.1097/MRR.0000000000000108 25603539 PMC4415968

[B45] Li J. Zhang Q. Li S. Niu L. Ma J. Wen L. (2020). alpha7nAchR mediates transcutaneous auricular vagus nerve stimulation-induced neuroprotection in a rat model of ischemic stroke by enhancing axonal plasticity. *Neurosci. Lett.* 730:135031. 10.1016/j.neulet.2020.135031 32416113

[B46] Li Y. Powers C. Jiang N. Chopp M. (1998). Intact, injured, necrotic and apoptotic cells after focal cerebral ischemia in the rat. *J. Neurol. Sci.* 156 119–132. 10.1016/s0022-510x(98)00036-7 9588846

[B47] Lin W. S. Chou C. L. Chang M. H. Chung Y. M. Lin F. G. Tsai P. Y. (2018). Vagus nerve magnetic modulation facilitates dysphagia recovery in patients with stroke involving the brainstem - A proof of concept study. *Brain Stimul.* 11 264–270. 10.1016/j.brs.2017.10.021 29162502

[B48] Lindemann J. Rakers C. Matuskova H. Simon B. J. Kinfe T. Petzold G. C. (2020). Vagus nerve stimulation reduces spreading depolarization burden and cortical infarct volume in a rat model of stroke. *PLoS One* 15:e0236444. 10.1371/journal.pone.0236444 32702055 PMC7377493

[B49] Liu A. F. Zhao F. B. Wang J. Lu Y. F. Tian J. Zhao Y. (2016). Effects of vagus nerve stimulation on cognitive functioning in rats with cerebral ischemia reperfusion. *J. Transl. Med.* 14:101. 10.1186/s12967-016-0858-0 27118204 PMC4847184

[B50] Liu J. Wang Y. Akamatsu Y. Lee C. C. Stetler R. A. Lawton M. T. (2014). Vascular remodeling after ischemic stroke: Mechanisms and therapeutic potentials. *Prog. Neurobiol.* 115 138–156. 10.1016/j.pneurobio.2013.11.004 24291532 PMC4295834

[B51] Liu T. T. Morais A. Takizawa T. Mulder I. Simon B. J. Chen S. P. (2022). Efficacy profile of noninvasive vagus nerve stimulation on cortical spreading depression susceptibility and the tissue response in a rat model. *J. Headache Pain* 23:12. 10.1186/s10194-022-01384-1 35062860 PMC8903561

[B52] Loerwald K. W. Borland M. S. Rennaker R. L. II Hays S. A. Kilgard M. P. (2018). The interaction of pulse width and current intensity on the extent of cortical plasticity evoked by vagus nerve stimulation. *Brain Stimul.* 11 271–277. 10.1016/j.brs.2017.11.007 29174302 PMC5898968

[B53] Long L. Zang Q. Jia G. Fan M. Zhang L. Qi Y. (2022). Transcutaneous Auricular Vagus Nerve Stimulation Promotes White Matter Repair and Improves Dysphagia Symptoms in Cerebral Ischemia Model Rats. *Front. Behav. Neurosci.* 16:811419. 10.3389/fnbeh.2022.811419 35493949 PMC9051615

[B54] Lu X. X. Hong Z. Q. Tan Z. Sui M. H. Zhuang Z. Q. Liu H. H. (2017). Nicotinic Acetylcholine Receptor Alpha7 Subunit Mediates Vagus Nerve Stimulation-Induced Neuroprotection in Acute Permanent Cerebral Ischemia by a7nAchR/JAK2 Pathway. *Med. Sci. Monit.* 23 6072–6081. 10.12659/msm.907628 29274273 PMC5747934

[B55] Ma J. Qiao P. Li Q. Wang Y. Zhang L. Yan L. J. (2019). Vagus nerve stimulation as a promising adjunctive treatment for ischemic stroke. *Neurochem. Int.* 131:104539. 10.1016/j.neuint.2019.104539 31445074

[B56] Ma J. Zhang L. He G. Tan X. Jin X. Li C. (2016). Transcutaneous auricular vagus nerve stimulation regulates expression of growth differentiation factor 11 and activin-like kinase 5 in cerebral ischemia/reperfusion rats. *J. Neurol. Sci.* 369 27–35. 10.1016/j.jns.2016.08.004 27653860

[B57] Ma J. Zhang L. Niu T. Ai C. Jia G. Jin X. (2018). Growth differentiation factor 11 improves neurobehavioral recovery and stimulates angiogenesis in rats subjected to cerebral ischemia/reperfusion. *Brain Res. Bull.* 139 38–47. 10.1016/j.brainresbull.2018.02.011 29432795

[B58] Manwani B. McCullough L. D. (2011). Sexual dimorphism in ischemic stroke: lessons from the laboratory. *Womens Health* 7 319–339. 10.2217/whe.11.22 21612353 PMC3128473

[B59] Martin C. O. Denburg N. L. Tranel D. Granner M. A. Bechara A. (2004). The Effects of Vagus Nerve Stimulation on Decision-Making. *Cortex* 40 605–612. 10.1016/s0010-9452(08)70156-4 15505970

[B60] Martino R. Foley N. Bhogal S. Diamant N. Speechley M. Teasell R. (2005). Dysphagia after stroke: Incidence, diagnosis, and pulmonary complications. *Stroke* 36 2756–2763. 10.1161/01.STR.0000190056.76543.eb 16269630

[B61] McIntire L. K. McKinley R. A. Goodyear C. McIntire J. P. Brown R. D. (2021). Cervical transcutaneous vagal nerve stimulation (ctVNS) improves human cognitive performance under sleep deprivation stress. *Commun. Biol.* 4:634. 10.1038/s42003-021-02145-7 34112935 PMC8192899

[B63] Morais A. Liu T. T. Qin T. Sadhegian H. Ay I. Yagmur D. (2020). Vagus nerve stimulation inhibits cortical spreading depression exclusively through central mechanisms. *Pain* 161 1661–1669. 10.1097/j.pain.0000000000001856 32142015 PMC7305968

[B64] Morrison R. A. Hulsey D. R. Adcock K. S. Rennaker R. L. II Kilgard M. P. Hays S. A. (2019). Vagus nerve stimulation intensity influences motor cortex plasticity. *Brain Stimul.* 12 256–262. 10.1016/j.brs.2018.10.017 30409712 PMC6347516

[B65] Nakka V. P. Gusain A. Mehta S. L. Raghubir R. (2008). Molecular mechanisms of apoptosis in cerebral ischemia: Multiple neuroprotective opportunities. *Mol. Neurobiol.* 37 7–38. 10.1007/s12035-007-8013-9 18066503

[B66] Nonis R. D’Ostilio K. Schoenen J. Magis D. (2017). Evidence of activation of vagal afferents by non-invasive vagus nerve stimulation: An electrophysiological study in healthy volunteers. *Cephalalgia* 37 1285–1293. 10.1177/0333102417717470 28648089 PMC5680905

[B67] Porter B. A. Khodaparast N. Fayyaz T. Cheung R. J. Ahmed S. S. Vrana W. A. (2011). Repeatedly Pairing Vagus Nerve Stimulation with a Movement Reorganizes Primary Motor Cortex. *Cereb. Cortex* 22 2365–2374. 10.1093/cercor/bhr316 22079923

[B68] Pruitt D. T. Schmid A. N. Kim L. J. Abe C. M. Trieu J. L. Choua C. (2016). Vagus Nerve Stimulation Delivered with Motor Training Enhances Recovery of Function after Traumatic Brain Injury. *J. Neurotrauma* 33 871–879. 10.1089/neu.2015.3972 26058501 PMC4860663

[B69] Redgrave J. Day D. Leung H. Laud P. J. Ali A. Lindert R. (2018a). Safety and tolerability of Transcutaneous Vagus Nerve stimulation in humans; a systematic review. *Brain Stimul.* 11 1225–1238. 10.1016/j.brs.2018.08.010 30217648

[B70] Redgrave J. N. Moore L. Oyekunle T. Ebrahim M. Falidas K. Snowdon N. (2018b). Transcutaneous Auricular Vagus Nerve Stimulation with Concurrent Upper Limb Repetitive Task Practice for Poststroke Motor Recovery: A Pilot Study. *J. Stroke Cerebrovasc. Dis.* 27 1998–2005. 10.1016/j.jstrokecerebrovasdis.2018.02.056 29580658

[B71] Roosevelt R. W. Smith D. C. Clough R. W. Jensen R. A. Browning R. A. (2006). Increased extracellular concentrations of norepinephrine in cortex and hippocampus following vagus nerve stimulation in the rat. *Brain Res.* 1119 124–132. 10.1016/j.brainres.2006.08.048 16962076 PMC1751174

[B72] Rychlicki F. Zamponi N. Cesaroni E. Corpaci L. Trignani R. Ducati A. (2006). Complications of vagal nerve stimulation for epilepsy in children. *Neurosurg. Rev.* 29 103–107. 10.1007/s10143-005-0005-5 16518639

[B73] Salvade A. Ryvlin P. Rossetti A. O. (2018). Impact of vagus nerve stimulation on sleep-related breathing disorders in adults with epilepsy. *Epilepsy Behav.* 79 126–129. 10.1016/j.yebeh.2017.10.040 29287215

[B74] Schmidt-Pogoda A. Bonberg N. Koecke M. H. M. Strecker J. K. Wellmann J. Bruckmann N. M. (2020). Why Most Acute Stroke Studies Are Positive in Animals but Not in Patients: A Systematic Comparison of Preclinical, Early Phase, and Phase 3 Clinical Trials of Neuroprotective Agents. *Ann. Neurol.* 87 40–51. 10.1002/ana.25643 31714631

[B76] Silberstein S. D. Yuan H. Najib U. Ailani J. Morais A. L. Mathew P. G. (2020). Non-invasive vagus nerve stimulation for primary headache: A clinical update. *Cephalalgia* 40 1370–1384. 10.1177/0333102420941864 32718243

[B77] Sucholeiki R. Alsaadi T. M. Morris G. L. III Ulmer J. L. Biswal B. Mueller W. M. (2002). fMRI in patients implanted with a vagal nerve stimulator. *Seizure* 11 157–162. 10.1053/seiz.2001.0601 12018958

[B78] Sun Z. Baker W. Hiraki T. Greenberg J. H. (2012). The effect of right vagus nerve stimulation on focal cerebral ischemia: An experimental study in the rat. *Brain Stimul.* 5 1–10. 10.1016/j.brs.2011.01.009 22037134 PMC3264742

[B79] Toffa D. H. Touma L. El Meskine T. Bouthillier A. Nguyen D. K. (2020). Learnings from 30 years of reported efficacy and safety of vagus nerve stimulation (VNS) for epilepsy treatment: A critical review. *Seizure* 83 104–123. 10.1016/j.seizure.2020.09.027 33120323

[B80] Tran Y. Shah A. K. Mittal S. (2011). Lead breakage and vocal cord paralysis following blunt neck trauma in a patient with vagal nerve stimulator. *J. Neurol. Sci.* 304 132–135. 10.1016/j.jns.2011.02.022 21397256

[B81] Vargas-Caballero M. Warming H. Walker R. Holmes C. Cruickshank G. Patel B. (2022). Vagus Nerve Stimulation as a Potential Therapy in Early Alzheimer’s Disease: A Review. *Front. Hum. Neurosci.* 16:866434. 10.3389/fnhum.2022.866434 35572001 PMC9098960

[B83] Wu D. Ma J. Zhang L. Wang S. Tan B. Jia G. (2020). Effect and Safety of Transcutaneous Auricular Vagus Nerve Stimulation on Recovery of Upper Limb Motor Function in Subacute Ischemic Stroke Patients: A Randomized Pilot Study. *Neural Plast.* 2020:8841752. 10.1155/2020/8841752 32802039 PMC7416299

[B84] Yang Y. Yang L. Y. Orban L. Cuylear D. Thompson J. Simon B. (2018). Non-invasive vagus nerve stimulation reduces blood-brain barrier disruption in a rat model of ischemic stroke. *Brain Stimul.* 11 689–698. 10.1016/j.brs.2018.01.034 29496430 PMC6019567

[B85] Yap J. Y. Y. Keatch C. Lambert E. Woods W. Stoddart P. R. Kameneva T. (2020). Critical Review of Transcutaneous Vagus Nerve Stimulation: Challenges for Translation to Clinical Practice. *Front. Neurosci.* 14:284. 10.3389/fnins.2020.00284 32410932 PMC7199464

[B86] Yuan H. Silberstein S. D. (2016). Vagus Nerve and Vagus Nerve Stimulation, a Comprehensive Review: Part I. *Headache* 56 71–78. 10.1111/head.12647 26364692

[B88] Yuan Y. Wang J. Wu D. Zhang D. Song W. (2019). Effect of Transcutaneous Vagus Nerve Stimulation in Dysphagia After Lateral Medullary Infarction: A Case Report. *Am. J. Speech Lang. Pathol.* 28 1381–1387. 10.1044/2019_AJSLP-18-0262 31498703

[B89] Zhang L. N. Zhang X. W. Li C. Q. Guo J. Chen Y. P. Chen S. L. (2021a). Vagal Nerve Stimulation Protects Against Cerebral Ischemia-Reperfusion Injury in Rats by Inhibiting Autophagy and Apoptosis. *Neuropsychiatr. Dis. Treat.* 17 905–913. 10.2147/NDT.S300535 33790559 PMC8008252

[B90] Zhang Y. Huang Y. Li H. Yan Z. Zhang Y. Liu X. (2021b). Transcutaneous auricular vagus nerve stimulation (taVNS) for migraine: An fMRI study. *Reg. Anesth. Pain Med.* 46 145–150. 10.1136/rapm-2020-102088 33262253

[B91] Zhao X. P. Zhao Y. Qin X. Y. Wan L. Y. Fan X. X. (2019). Non-invasive Vagus Nerve Stimulation Protects Against Cerebral Ischemia/Reperfusion Injury and Promotes Microglial M2 Polarization Via Interleukin-17A Inhibition. *J. Mol. Neurosci.* 67 217–226. 10.1007/s12031-018-1227-7 30484061

